# Route choice estimation in rail transit systems using smart card data: handling vehicle schedule and walking time uncertainties

**DOI:** 10.1186/s12544-022-00558-x

**Published:** 2022-07-19

**Authors:** Thomas James Tiam-Lee, Rui Henriques

**Affiliations:** INESC-ID, Instituto Superior Técnico, Universidade de Lisboa, Lisbon, Portugal

**Keywords:** Urban rail transit, Transportation, Statistical modelling, Route choice

## Abstract

Several cities around the world rely on urban rail transit systems composed of interconnected lines, serving massive numbers of passengers on a daily basis. Accessing the location of passengers is essential to ensure the efficient and safe operation and planning of these systems. However, passenger route choices between origin and destination pairs are variable, depending on the subjective perception of travel and waiting times, required transfers, convenience factors, and on-site vehicle arrivals. This work proposes a robust methodology to estimate passenger route choices based only on automated fare collection data, i.e. without privacy-invasive sensors and monitoring devices. Unlike previous approaches, our method does not require precise train timetable information or prior route choice models, and is robust to unforeseen operational events like malfunctions and delays. Train arrival times are inferred from passenger volume spikes at the exit gates, and the likelihood of eligible routes per passenger estimated based on the alignment between vehicle location and the passenger timings of entrance and exit. Applying this approach to automated fare collection data in Lisbon, we find that while in most cases passengers preferred the route with the least transfers, there were a significant number of cases where the shorter distance was preferred. Our findings are valuable for decision support among rail operators in various aspects such as passenger traffic bottleneck resolution, train allocation and scheduling, and placement of services.

## Introduction

Public urban rail transit is a major mode of transportation in many cities around the world. The latest report by the International Association of Public Transport estimates that metro systems had a total global ridership of 53,768 million in 2017, with Asia-Pacific and Europe leading the numbers [20]. The massive usage of urban rail transit underscores the importance of government efforts to ensure that metro systems are reliable, safe, and efficient for the public. Among the challenges tackled by metro operators is the effective resolution of bottlenecks in passenger traffic, including insufficient capacity for passenger demand, a task that has even more relevance in the context of the current COVID-19 pandemic to ensure the satisfaction of health safety norms.

Passenger route choices are not deterministic as they depend on the subjective perception of travel time, required transfers, convenience factors, and on-site train arrivals and waiting times, among others [5]. This makes it difficult to infer the volume of passengers along specific segments of the network at a given time.

In this study, we model individual passenger routes and the overall passenger flow in a rail network based only on automated fare collection data containing passenger entrances and exits within the urban rail system. To accomplish this task, we present a computational approach that assesses the likelihood of each possible route choice by aligning card validation timestamps against real-time route scheduling. In the absence of vehicle geolocation data, the locations of the trains at different times can be estimated by analyzing passenger volume peaks at the exit station gates. We can then use this information together with an analysis of the trip durations to better infer the likelihood that a passenger took a specific route by analyzing the location and timings of his or her entry and exit from the urban rail system.

In our approach, train arrival times are estimated by aligning passenger volume peaks at exit stations based on the theoretical velocity of the train along each line. Furthermore, our approach considers the variability of waiting times as a result of missing the next available train. It can be implemented without the use of complex and privacy-invasive sensors and monitoring devices and is adaptable to changes in the network not only because of unforeseen events like malfunctions and delays, but also changes in operational schedules and policies. This makes it robust to short-term changes in train dispatch times.

In this paper, we apply the proposed approach on the automated fare collection dataset of the Lisbon Metro for the month of October 2019 and discuss the corresponding results. The paper is structured as follows. Section 2 contains a discussion of related work. Section 3 contains a brief background of the Lisbon Metro and the automated fare collection dataset used in this paper. Section 4 introduces the proposed approach for estimating passenger route choices and passenger flow. Section 5 shows the results and validation of the proposed approach, and Sect. 6 contains the concluding remarks.

## Related work

This section discusses relevant advances on the task of inferring passenger route choice behavior within urban rail systems and the position of our work among these contributions. Traditionally, passenger route preferences are obtained through surveys which are then modelled mathematically to provide a systematic view of the passenger flow in a network [7]. In this approach, passenger preferences are usually modelled in accordance with several parameters such as in-vehicle travel time, transfer time, in-vehicle crowding, among others [15, 16]. Prato [14] surveys mathematical models used in modelling route choice behavior. Despite the relevance of this class of approaches, a core disadvantage of these approaches is the impracticality of collecting on-the-ground passenger preference data, which is both time-consuming and costly. Furthermore, dynamic behavior and systemic changes are difficult to capture with the static nature of the data on which the models are based upon. In the work of Zhu et al. [24], a method has been proposed to calibrate existing models with more empirical data.

The global rapid adoption of automated fare collection (AFC) systems triggered new opportunities. AFC data acquisition can address the key limitations of surveys, providing dynamic information on passenger behavior. Sun et al. [17] estimates the density of in-vehicle and waiting rail passengers based on passenger entrance and exit times on a single segment of a rail network. Train velocities and dwell times are estimated using a linear regression model from the minimum travel times of a passenger for each pair of stations. Kim et al. [9] studied passenger preferences in taking regular versus express trains by inferring the passenger’s preference from travel time and train schedule. A limitation of the above studies is their restricted applicability to single lines, not capturing the variability of route choices in complex rail networks with interconnecting lines and stops.

Nonetheless, some works have proposed approaches to infer passenger choices in the context of multiple-line rail systems. Kusakabe et al. [10] infers the train type choices of passengers by enumerating the possible paths each passenger takes based on train timetables. While still technically operating on a single route, this work considers the possibility of switching train types (e.g., rapid, express) in the middle of the journey, which is conceptually equivalent to the multiple-line scenario. However, it places an assumption that the passengers always minimized their travel time first, then the number of transfers second, which may not always be the case in real-world situations due to both personal and external factors. Hong et al. [8] targets passenger route choices in interconnected lines. They estimate boarding and alighting time intervals of passengers based on entry and exit timings of the most efficient passengers and the arrival and departure times of the trains. From this estimation, each passenger is matched into the appropriate boarding and alighting group in order to determine the passenger’s most likely route in the network. Wu et al. [21] used a clustering approach to group the travel times of passengers for a particular origin and destination station. Thereafter, for each possible route, theoretical travel times from the origin station to the destination station are computed from the train schedules, allowing the estimation of walking and transfer times. Finally, the theoretical travel times are matched to the corresponding cluster centers to determine the route taken by each passenger.

While the above studies can consider multiple routes, they still present some limitations. First, they work under the assumption of well-defined train timetables as well as the punctuality of the trains. While in some major cities, such as Tokyo and Beijing, it is common to have precise train schedules that are followed on the dot, this is not always the case in many other cities [3, 4, 11]. Second, they do not consider the waiting time of passengers due to external factors and circumstances. For example, it is possible for passengers to miss a train because they were waiting for a friend, or the train was too crowded for their liking. In several of the aforementioned studies, transfer times and waiting times for each station are modeled as static parameters, thus disregarding those possibilities.

There are two studies that propose more complex models for the variability of waiting and transfer times. In the work of Sun and Xu [18], a generalized concept of the platform waiting time called platform-elapsed time, which comprised of the actual waiting time and any additional delays caused by missing the trains. The platform-elapsed time is modelled as a random variable following a geometric distribution. Similarly, in the work of Zhao et al. [23], the number of trains skipped at transfer stations is modelled as a random variable following a polynomial distribution. However, there are still two caveats. First, the parameters of the distribution are estimated based on the train arrival times, which as mentioned are not always precise in all rail systems. Second, there is an assumption that waiting time distributions are consistent.

Finally, a few studies target the task of inferring passenger route choices with the help of mobile phone trajectories [19, 22]. These studies augment the inference of route choices with additional information obtained from passengers’ mobile phones. Yet, such principles may not be easily applicable by the transport operators and other stakeholders as mobile data is not always easily accessible due to poor connectivity in subway systems as well as privacy and ethical concerns.

Our proposed method for inferring passenger route choices offers several advantages compared to previous approaches. First, it requires minimal information that is easily obtainable in most urban rail systems. Specifically, aside from the automated fare collection data, our approach does not require precise timetables that define arrival and departure times for each station. Furthermore, our approach also considers the variability of waiting times as a result of missing the next available train. The proposed approach can be implemented without the use of complex and privacy-invasive sensors and monitoring devices and is adaptable to changes in the network not only because of unforeseen events like malfunctions and delays, but also changes in operational schedules and policies. Table 1 summarizes the contributions of our work in relation to previous studies in inferring passenger routes in urban rail systems. Among the studies on individual passenger route inference, our approach is to our knowledge the only one that does not require precise train timetables.

**Table 1:** Studies on inferring passenger route choice behavior in urban rail systems

Study	Single line?	Multiple lines?	Applicable to route choice estimation?	Considered skipping/missing trains?	Precise train timetables?	Additional sensors?
Sun et al. [17]	Yes	No	No	Yes	Not required	Not required
Kim et al. [9]	Yes	No	No	No	Not required	Not required
Tao et al. [19]	Yes	Yes	No	No	Not required	Mobile signal
Kusakabe et al. [10]	Yes	Yes	Yes	No	Required	Not required
Hong et al. [8]	Yes	Yes	Yes	No	Required	Not required
Wu et al. [21]	Yes	Yes	Yes	Yes	Required	Not required
Sun and Xu [18]	Yes	Yes	Yes	Yes	Required	Not required
Zhao et al. [23]	Yes	Yes	Yes	Yes	Required	Not required
Our approach	Yes	Yes	Yes	Yes	Not required	Not required

## Case study

### Problem formulation

The objective of this work is to estimate the route choices of passengers in a mass transit system, in which the system is composed of a set of interconnected lines, with each line serviced periodically by vehicles passing through a series of stations. Passengers may enter and exit the system through any station and may take as vehicles as many times as they want while within the network. We define the route of a passenger as the path taken from the station of entry to the station of exit. We then formalize the task as follows: given the passengers’ time and location of entry and exit in the transit system, how can we predict the actual routes that were taken by each passenger?

### Lisbon metropolitan system

**Fig. 1:**
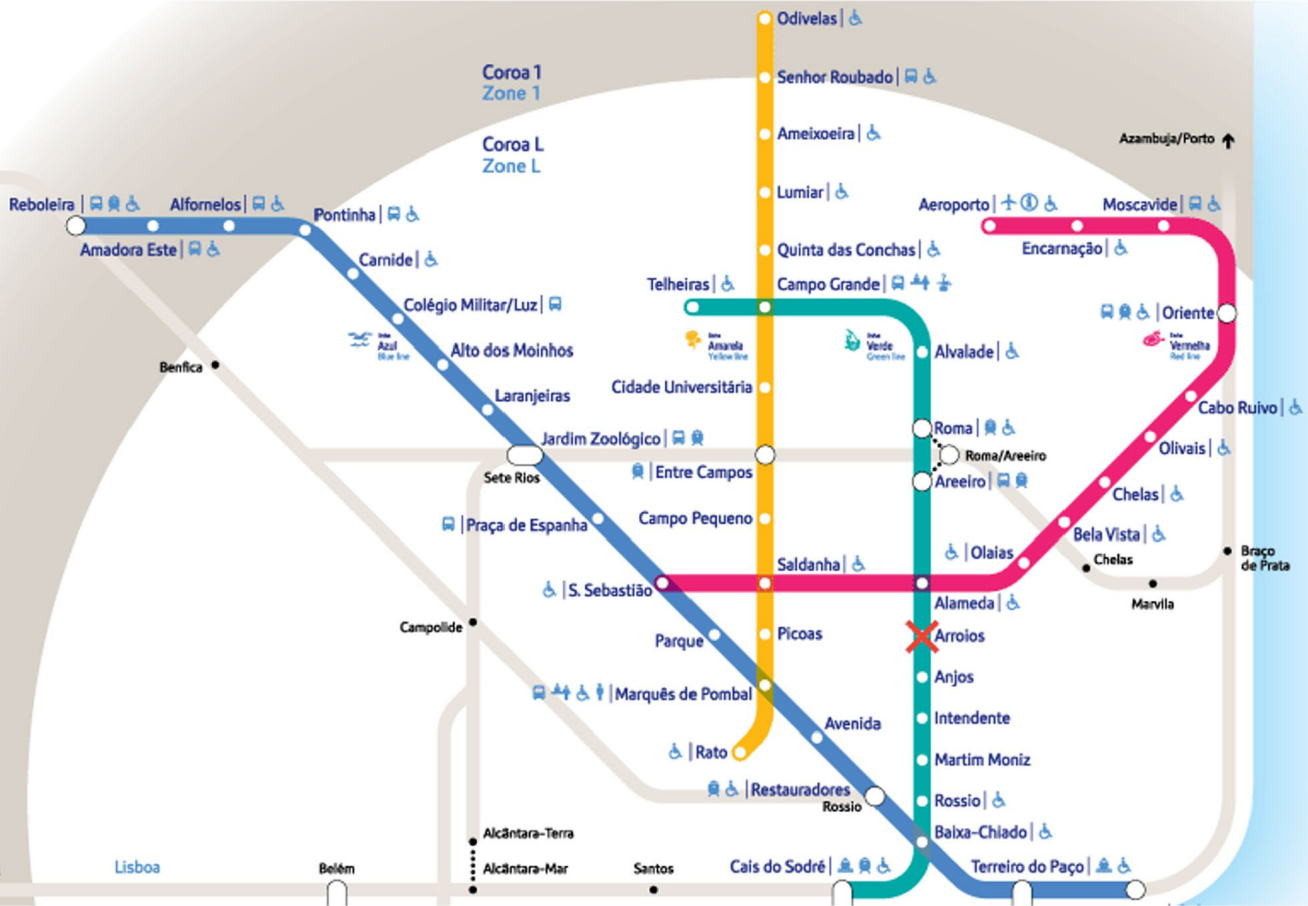
Lisbon metro network map

In this work, we consider the Lisbon metropolitan system, also referred as Metro, as the guiding case study to assess passenger route choice estimators. The Metro is an urban rail rapid transit system in Lisbon, the capital city of Portugal. As of 2021, it consists of 4 lines: *azul* (blue), *amarela* (yellow), *verde* (green), and *vermelha* (red) with a total of 56 stations [12]. As with most other urban rail transit systems, the different lines interconnect with one another through certain common stations. Figure 1 shows a map of the rail network.

As the rapid transit system of a capital city, the Lisbon Metro serves a massive number of passengers. In 2019, the annual ridership of the Lisbon Metro reached 173 million people [13]. Similar with most other urban rail systems, the Lisbon Metro makes use of an automated fare collection system, in which passengers use cards to enter and exit the stations. After entering, the passenger is free to ride any train within the network as many times as they want, before exiting at any station. With each entrance and exit made by each passenger, the automated fare collection system records the following information: the timestamp (date and time), the station, and a unique identifier for the passenger (i.e., the number of the card used for the entry or exit). The collection of all such records from the automated fare collection system forms the dataset that we will be using for estimating passenger flow and route choices within the network. Specifically, we will be focusing on data for the month of October 2019 containing over 33 million entrances and exits.

## Methods

In this section, we present a two-step approach for estimating passenger route choices in urban rail transit systems. The approach is divided into two parts: (a) estimating the locations of the trains in the network, and (b) estimating the likelihood of each passenger’s route choice based on the train locations.

### Estimation of the train locations

The first step is to estimate the locations of each train in the network over time. To this end, peaks in the volume of passengers exiting the stations are identified based on automated fare collection data. For any given day, we can form a time series for each station by aggregating the number of exit records per station into 15 s intervals. We then perform a rolling average and rolling deviation on the time series to identify passenger exit peaks along a line to estimate the precise location of vehicles. We define a passenger volume spike as a peak in the time series that is at least one standard deviation higher than rolling mean. This scheme aims to ensure that only peaks deviating from typical behavior along a given period are considered and has previously been used similarly for robust peak detection in other studies [2, 6].

**Fig. 2:**
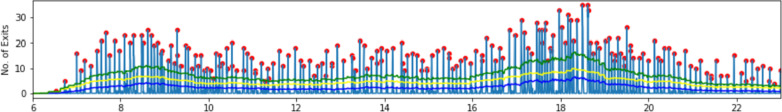
Volume of passengers over time for October 7 in Santa Apolónia station. Red dots show spikes in passenger volume, blue line shows the rolling mean, yellow line shows the rolling standard deviation, and green line shows one standard deviation away from the rolling mean

Using the Lisbon Metro as an example, Fig. 2 shows the aggregated passenger exits on Santa Apolónia station on a single day. The red dots represent the passenger volume spikes, as identified through the peaks that are at least one standard deviation away from the rolling mean. From this, it is apparent that there are periodic spikes in the number of people exiting the station, caused by groups of people alighting from the trains that arrive periodically. We identify such spikes in passenger volume and use them as a basis for estimating the train locations within the network.

We then identify the travel time durations of each train through each line. This data can easily be obtained based on the urban rail system’s operational protocols or, in the absence of this data, could be estimated from averaging times of actual train operations. Given a line consisting of stations $s_1,s_2,...,s_n$, the train arrival offset at station $s_i$, referred to as $offset(s_i)$, is the total amount of time it takes for a train to arrive at station $s_i$ from station $s_1$. Thus, $offset(s_i)$ can be defined recursively, where $offset(s_1)=0$ and $offset(s_i)=offset(s_{i-1})+\delta (s_{i-1},s_i)$, with $\delta (s_{i-1},s_i)$ being the time takes for the train to arrive to $s_i$ from $s_{i-1}$. Note that the train arrival’s offsets on the same line moderately differ from the reverse direction.

**Fig. 3:**
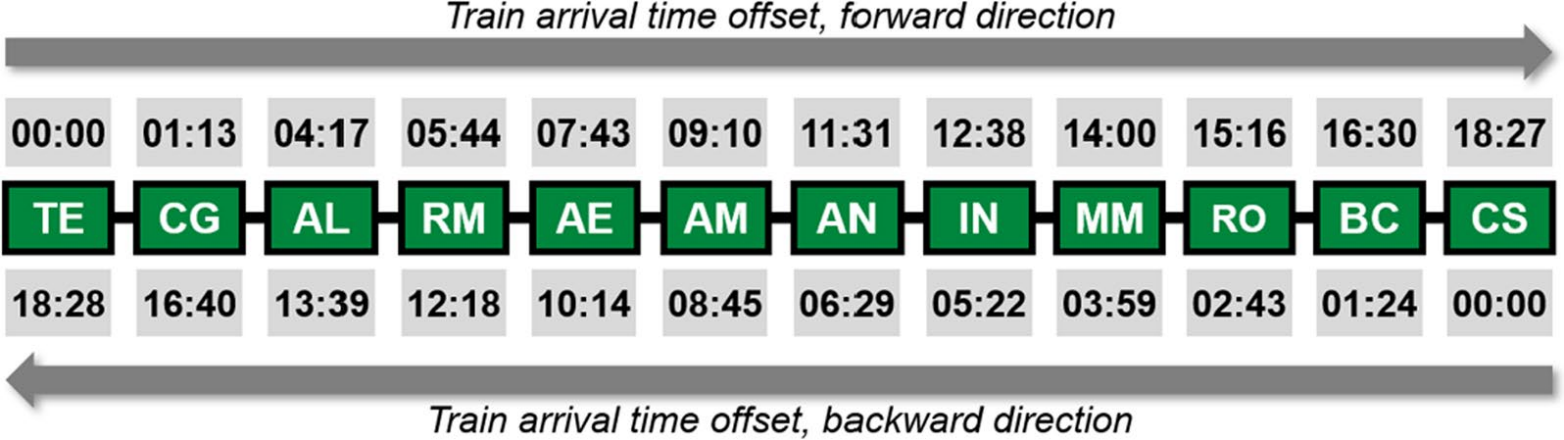
Train arrival time offsets from the first station in *Linha Verde* (green line)

Using the Lisbon Metro’s green line as an example, Fig. 3 shows the train arrival offsets of each station for both directions.

The next step is to align the passenger volume peak data for each station according to the projected passing of the train along the stations using the previously identified offsets. To do this, we perform a shift by subtracting the corresponding train arrival offset from each of the passenger volume peaks in each station. We then estimate the likelihood that a train started on the first station at a given time. To this end, we define a likelihood score based on the total distance from the closest passenger volume spikes. Given a starting time *t*, the likelihood score ${\mathcal {L}}(t)$ is computed as:

1 $$\begin{aligned} {\mathcal {L}}(t)=1-\frac{1}{\alpha ^2}\sum _{s \in S}\frac{\left( closest(s,t,\alpha )-t\right) ^2}{count(s,t,\alpha )+1} \end{aligned}$$

where *S* is the set of stations in the target time and $\alpha$ is a constant time duration threshold. The *closest* function returns the time of the closest passenger volume peak (after shifting) that occurs after *t*, bounded by a maximum value of $t + \alpha$. The *count* function returns the total number of exits made within the range $\left[ t-\alpha ,t+\alpha \right]$ (after shifting). In this work, $\alpha =180$ by default, but can be adjusted accordingly. Intuitively, the numerator represents the squared error of the train arrival times for each station from the closest passenger volume peak, while the denominator weighs the errors according to the volume of passengers to give more importance to stations with larger volumes of passengers around that time. The 1 constant is added to ensure that the operation is defined even in the absence of passenger exits. Finally, the summation of the errors is normalized to the range $\left[ 0,1\right]$, where 1 represents a perfect alignment of the train arrivals with passenger volume peaks. We can identify the times where trains are likely to have started from the first station of the line by identifying the peaks of the likelihood score plot.

**Fig. 4:**
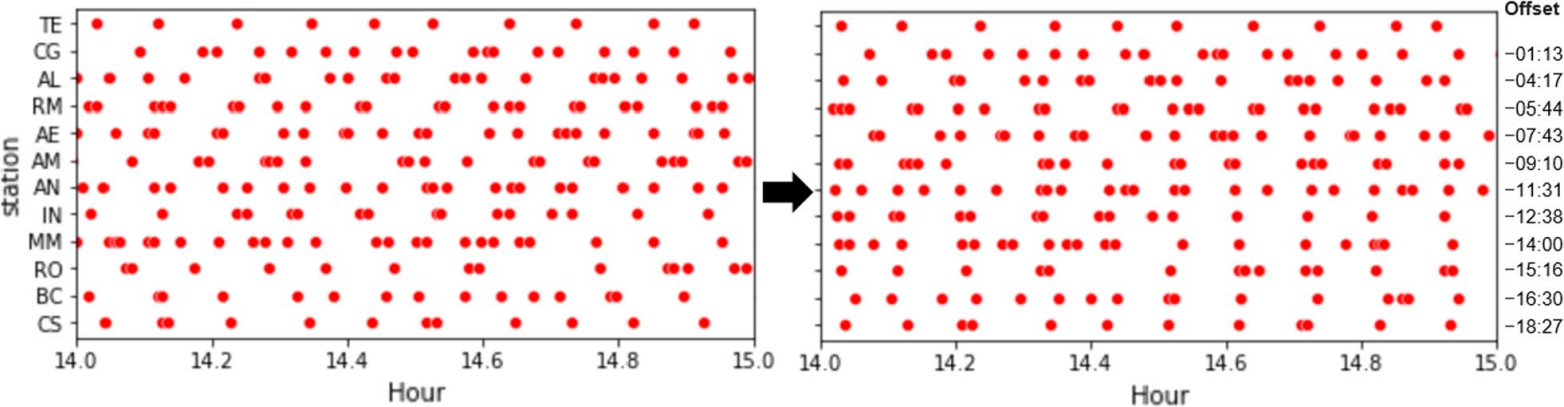
Passenger volumes spikes per station on *Linha Verde* (green line) before and after shifting according to train arrival time offsets

**Fig. 5:**
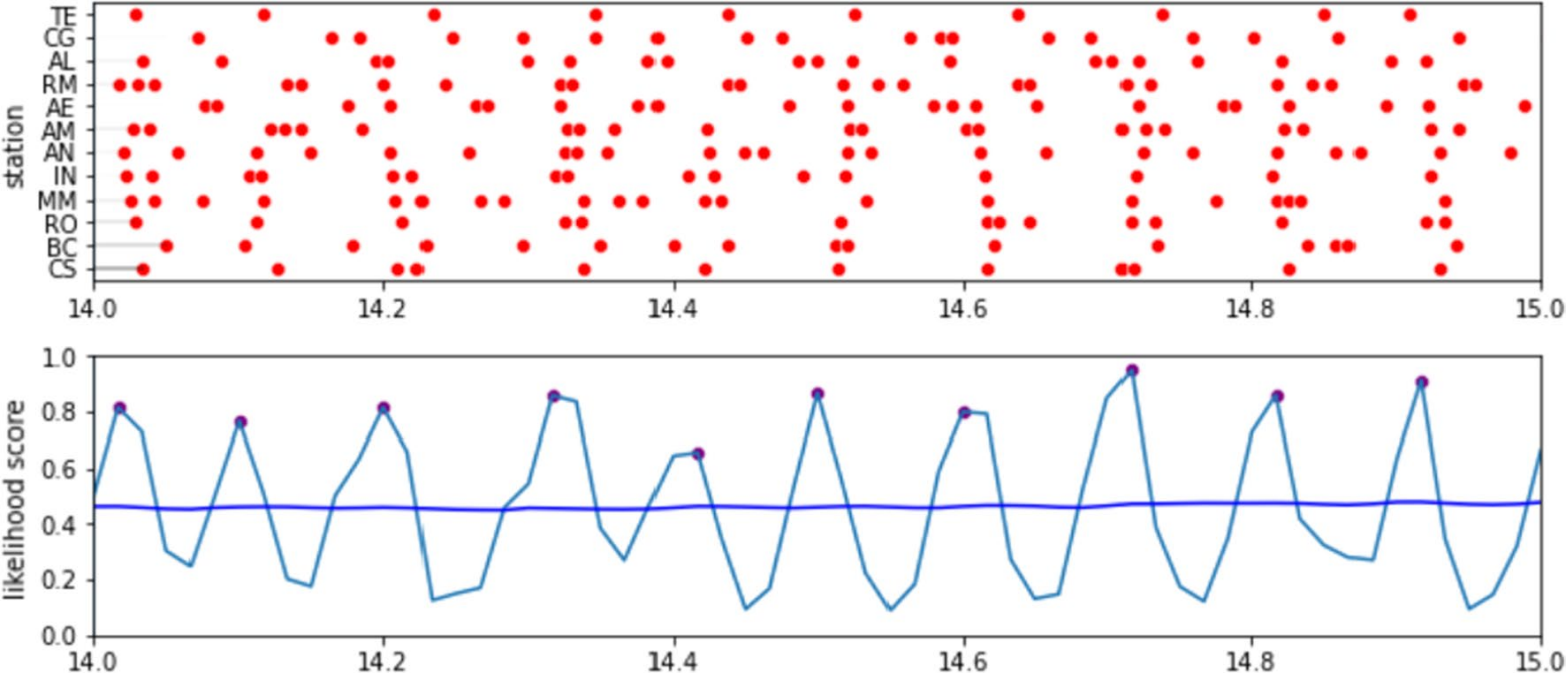
Likelihood score computed across different times, showing the score peak at times where the passenger volume peaks are well-aligned

Illustrating, Fig. 4 shows the times of the passenger volume spikes in *Linha Verde* (green line) before and after shifting. Based on our assumption that the arrival of a train on a station implies a possible spike in passenger volume at the exit gates, we are able to draw a vertical line on the shifted plot and observe volume spikes close to that line. We can visually observe in the figure that this hypothesis holds true, particularly on the latter half of the line. This makes sense because people are more likely to get off at later stations according to the natural flow of the line direction. Figure 5 shows ${\mathcal {L}}(t)$ computed for various values of *t*, showing the likelihood peaks at times where the passenger volume peaks are well-aligned, with the purple dots showing the peaks which represent the predicted times that the trains have started travelling from the first station in this direction.

Now that the predicted starting times of the trains have been identified, actual train arrival times for each station can be easily inferred. To compute the train arrival times at a given station $s_i$, we simply add $offset(t_i)$ to each of the estimated starting times from the lines passing through $s_i$. Using this information, given a passenger’s entrance and exit information from the automated fare collection data, potential routes taken from entrance to exit can now be explored.

### Prediction of passenger route choices

Before route choices are predicted, we first pair the entrance and exit records in the automated fare collection dataset. Given that each record contains the following information: (a) entrance or exit, (b) timestamp, (c) station, and (d) identifier, we can match each passenger’s entrance record with its corresponding exit record considering the card identifiers and trip precedencies. There may be some cases where an entrance or exit record is missing its corresponding pair in the dataset. These can occur due to operational anomalies (e.g., malfunctions, cases where passengers were allowed to enter/exit without passing through the gates in extraordinary situations). Understandably, route choice estimation for incomplete trips should be proceeded by well-established principles for boarding or alighting station inference [1]. Nevertheless, the likelihood of incomplete trip records for rail systems with closed gates is generally low.

**Fig. 6:**
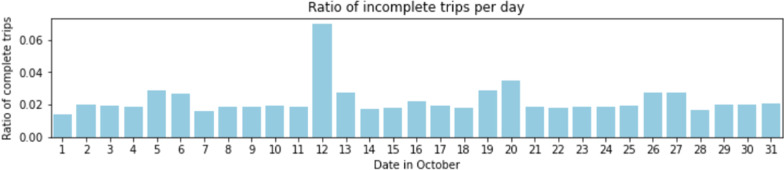
Ratio of incomplete trips over all trips per day in the Lisbon Metro

In fact, after pairing the entrance and exit records in the Lisbon Metro, Fig. 6 shows the ratio of incomplete trips (i.e., missing entrance or missing exit) over all trips for each day in the month of October. Overall, only 2.09% of the trips are incomplete, although there was an unusually high number of incomplete trips on the 12th of the month, likely caused by an unusual operational event. To remove the additional uncertainty associated with route choices along incomplete trip records, we excluded these records from the conducted experimental analysis.

We define a route as a path that a passenger takes to get from an entry to an exit station. A route may contain one or more segments, which represent a single train ride on any given line. Given a passenger’s time and location of entry and a candidate route, the expected exit time of the passenger by following that route can be computed. To estimate the expected exit time along a route, travel can be simulated considering the train arrival times for the relevant stations.

To accommodate for cases where a passenger misses the next available train (e.g., train is full, walked too slowly from gate to the train platform, waiting for a friend), we introduce a parameter $max\_lag$ which represents the maximum number of trains skipped per segment of the route. Thus, for each route the algorithm returns a set of expected exit times, each computed based on the number of trains skipped in different segments of the route. To formalize, given the time of entrance and a candidate route, we estimate the time of exit using Algorithm 1.
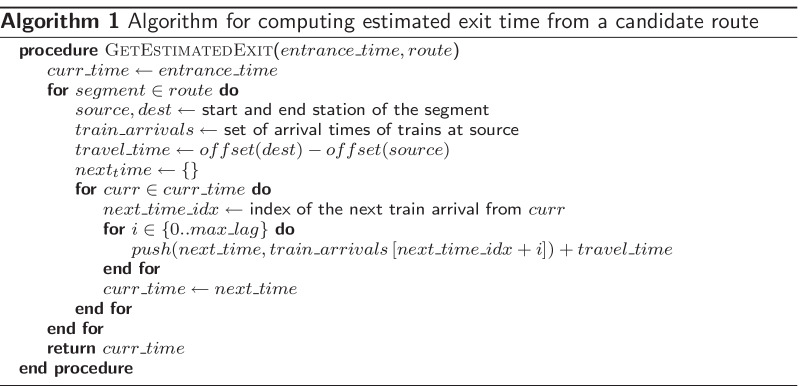


Given a set of estimated exit times $R=\{r_1,r_2,...\}$ from a specified route and the actual exit time $t_{exit}$ of the passenger, an error score can be computed as $\min _{r_i \in R}(r_i-t_{exit})^2$. The error scores of different candidate routes could be compared to assess the likelihood that each route was taken, with a smaller score representing a better likelihood. A threshold can also be placed to ensure that the error is small enough to assert confidence in the prediction.

**Fig. 7:**
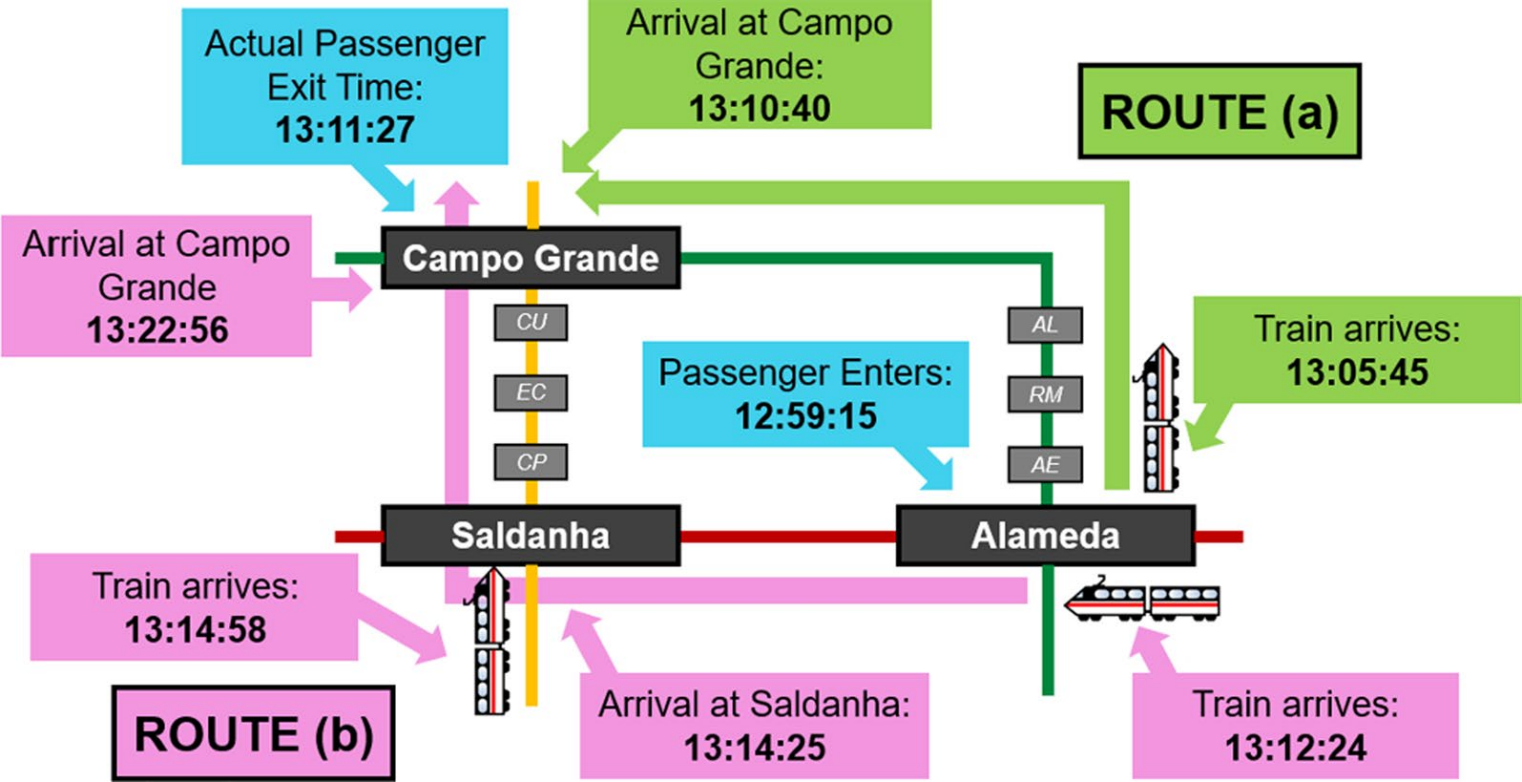
Two possible routes from Alameda to Campo Grande, shown in light green (**a**) and pink (**b**). It is more likely that the passenger took route (**a**) based on the distance between the expected arrival at Campo Grande and the actual exit time

In the Lisbon Metro dataset, consider for instance a passenger who entered through Alameda station at 12:59:15 and exited through Campo Grande station at 13:11:27. Two possible routes that this passenger could have taken are as follows: (a) green line from Alameda to Campo Grande and (b), red line from Alameda to Saldanha, followed by the yellow line from Saldanha to Campo Grande. As visualized in Fig. 7, from the time of entry, the expected exit time if route (a) was taken is 13:10:40, while the expected exit time if route (b) was taken is 13:22:56. Since the actual duration of the passenger’s trip was 13:11:27, it is more likely that route (a) was taken.

**Fig. 8:**
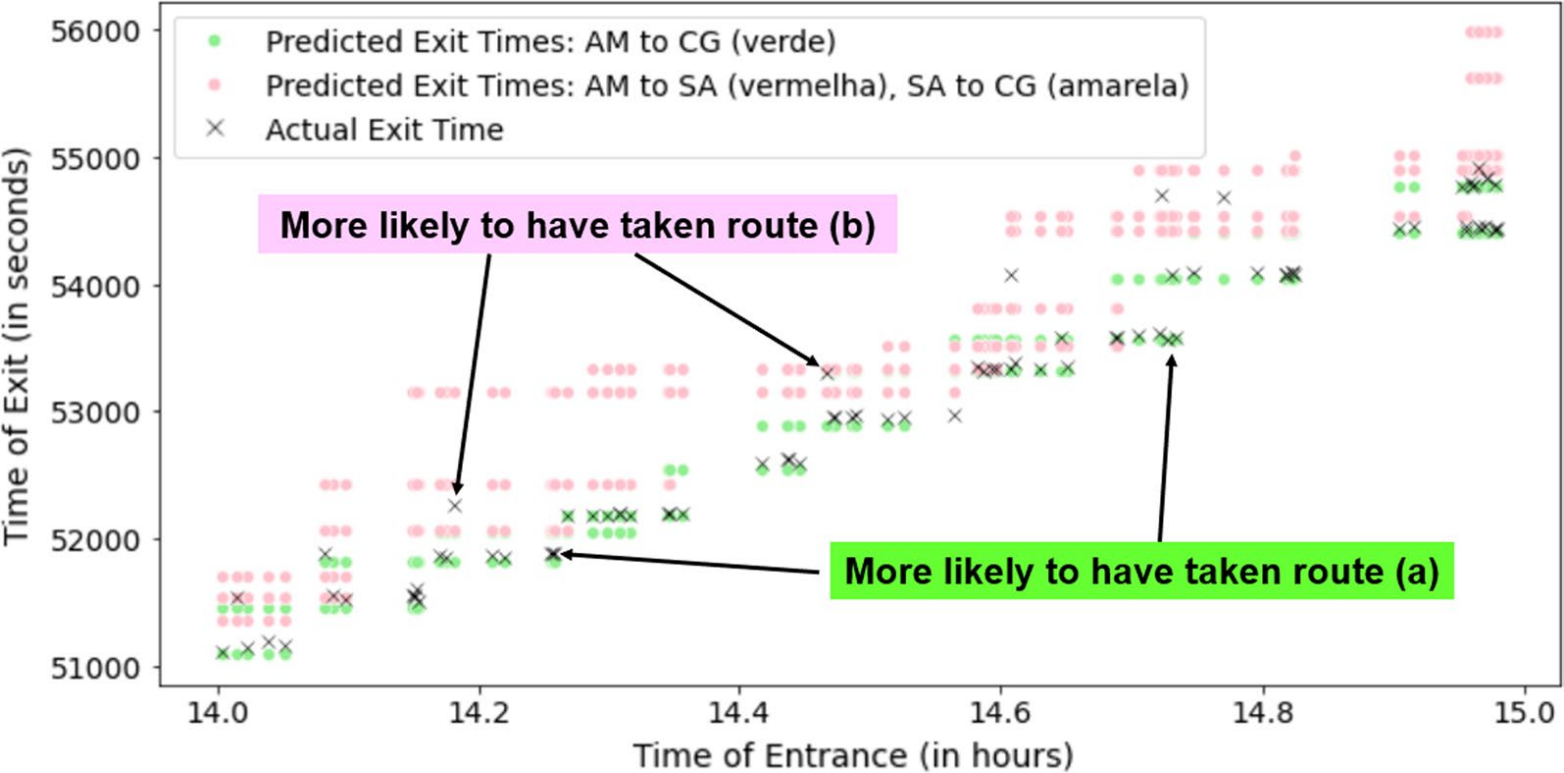
Actual passenger exit times in comparison with the expected exit times for two different routes from Alameda to Campo Grande

In Fig. 8, we can see a plot of the actual exit times of each passenger who entered through Alameda and exited through Campo Grande, as well as the expected exit times of route (a) in light green and route (b) in pink, both computed with a $max\_lag$ of 1. Most passenger exits are closer to the expected exit times of route (a), which is the more direct path that does not require any transfers. Nevertheless, it can be seen that there are a few passengers likely to have taken the other route based on their exit timing.

## Results and discussion

To validate the approach, the introduced methodology was applied to the Lisbon Metro dataset. For simplicity, the reported results are conducted on a single randomly selected day for the purposes of this analysis: October 7, 2019, which contains 600,548 pairs of entrance-exit data. While the absence of ground-truth data on passenger preferences makes it infeasible to provide an absolute comparison, we make use of available knowledge to check the consistency of the results of the approach against background expectations.

**Fig. 9:**
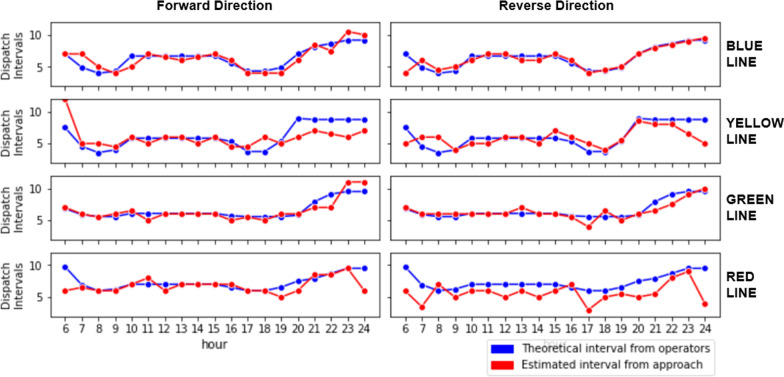
Average train dispatch intervals: comparison of estimated and theoretical values across different lines and hours of the day

A key component of the proposed approach is the estimation of train dispatch times in the absence of precise timetables. However, in the case of Lisbon Metro, while precise timetables are not available, approximate waiting times for each of the four lines are made available to the public for different times of the day.^1^ As such, it is possible to validate the estimated train dispatch intervals of the proposed approach against the theoretical dispatch frequencies. Figure 9 shows the average dispatch intervals across different hours of the day.

It can be seen that estimated dispatch intervals mostly coincide with the theoretical intervals defined by the Lisbon Metro for each of the four lines. There are a few noticeable deviations towards the end of the day, which could be attributed to abnormal peak patterns due to sparse ridership. Nonetheless, the overall consistency of the estimated train dispatch intervals with the theoretical dispatch intervals shows the possibility of estimating train locations, without precise timetables, from passenger volume peaks.

To validate the prediction of passenger routes, we exploit the case where there is only one possible route from the entrance and exit station, and that route is without any transfers, i.e. station pairs on the same segment of the line without branching paths. In these cases, we assume that the passenger *must* have taken the undoubtedly shortest route. For these cases, we run the algorithm to validate whether the predicted exit times of the only available route are reasonably close to the actual exit times of the passengers. We used a $max\_lag$ of 1, assuming that most passengers will not skip trains apart from cases passenger did not walk fast enough to the platform to catch the next available train.

**Fig. 10:**
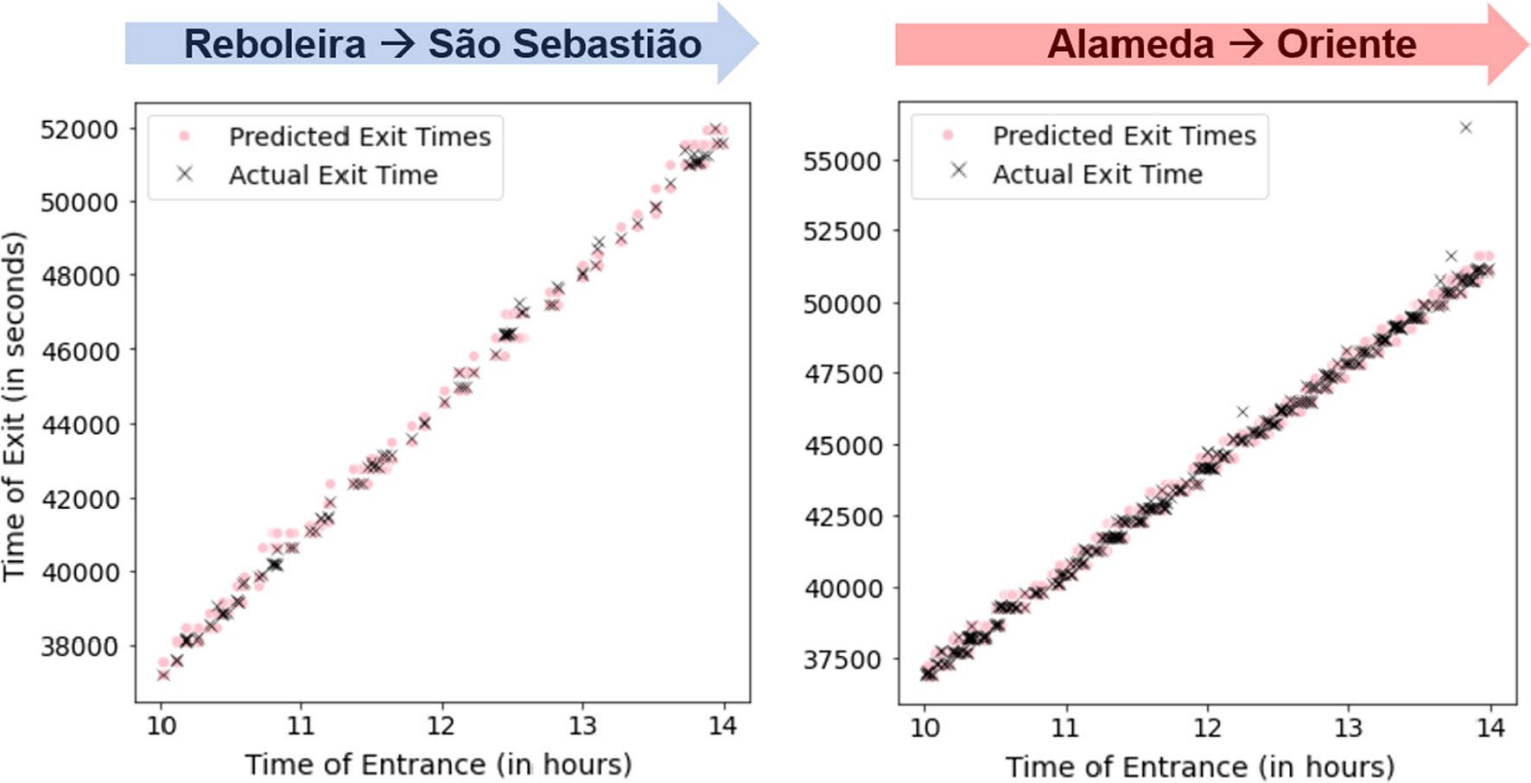
Examples of two single-route pairs to validate the prediction of passenger exit times

Illustrating, Fig. 10 shows two such examples: Reboleira $\rightarrow$ São Sebastião from the Blue Line and Alameda $\rightarrow$ Oriente from the Red Line. In both of these pairs, there is only one possible route and as such we assume that passengers should have taken that route. We can see that visually, this is indeed the case, apart from a few cases in the Alameda $\rightarrow$ Oriente route where the actual exit time was considerably later than the predicted exit times. These cases could be either motivated by the fact that a passenger skipped more than one available train or special cases (e.g., individuals with lengthy stops at metro stores and *cafes*, subway workers). Nonetheless, these cases are a minority and most passenger exit times are accurately predicted.

Next, we look at cases where multiple route choices are involved. To do this, we first identify all pairs of stations within the rail network. For each pair $(s_a,s_b),s_a \ne s_b$, we identify all candidate route choices from station $s_a$ to $s_b$ though a standard exhaustive graph search algorithm. To limit the candidate route choices, we impose the restriction that a route cannot contain the same station more than once. We then apply the proposed approach to predict the route that is more likely to have been taken by each passenger who entered through $s_a$ and exited through $s_b$. We used $max\_lag=1$, $\alpha =180$, an error threshold of $180^2=32400$ to only consider predictions that are within 180 s of the closest expected exit time. Because of this, not all passenger trips are assigned a predicted route choice, i.e., if the exit time is not within 180 s of the expected exit times of the candidate routes, the route choice is deemed “unknown”. This is to be expected given the presence of small markets, stores and coffee shops within a few stations in the network. In the dataset, 89% of the trips were successfully assigned to a candidate route choice (i.e., not “unknown”).

In our analysis, we highlight two common criteria for passenger preference: (a) “least transfers” (least number of transfers between lines, a measure of convenience and time), and (b) “least stations” (least number of stations passed, a measure of distance and time). There are a total of 49 stations across all lines in the metro, resulting in 2352 possible entrance-exit pairs. Out of these pairs, 636 are non-trivial entrance-exit pairs where the route with the least transfers is different from the route with the least stations.

Among the 636 non-trivial station pairs, the preferred route is the one with least transfers in 562 (88.36%) of the pairs. On the other hand, the preferred route is the one with the least number of stations in 54 (8.49%) of the pairs. As for the remaining pairs, the preferred route was neither of the two. It is possible to visualize the preference of passengers for various pairs of stations.

**Fig. 11:**
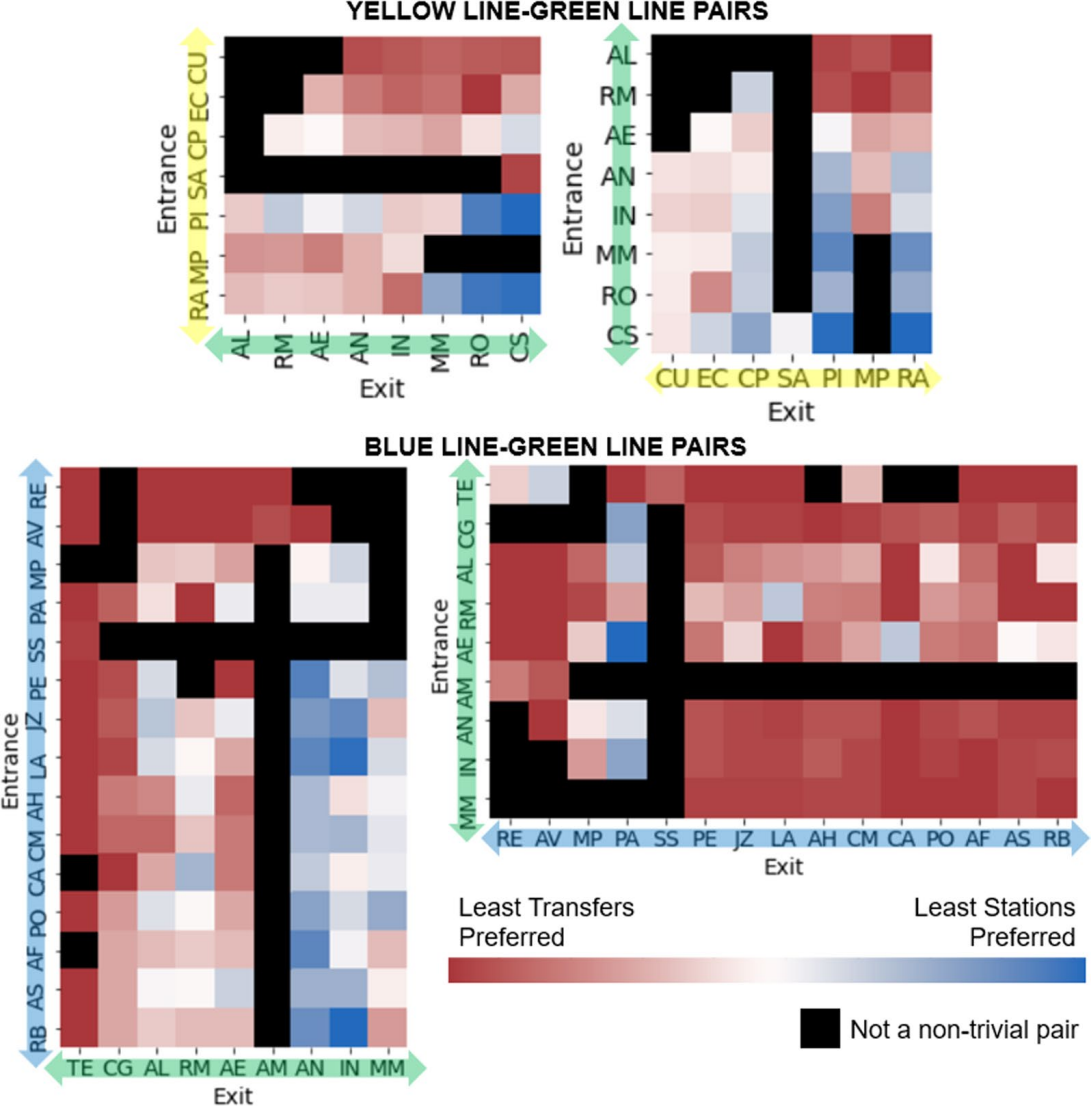
Route preference for passengers on non-trivial pairs in the yellow-green lines and blue-green lines. For conciseness, rows and columns with no non-trivial pairs are omitted

**Fig. 12:**
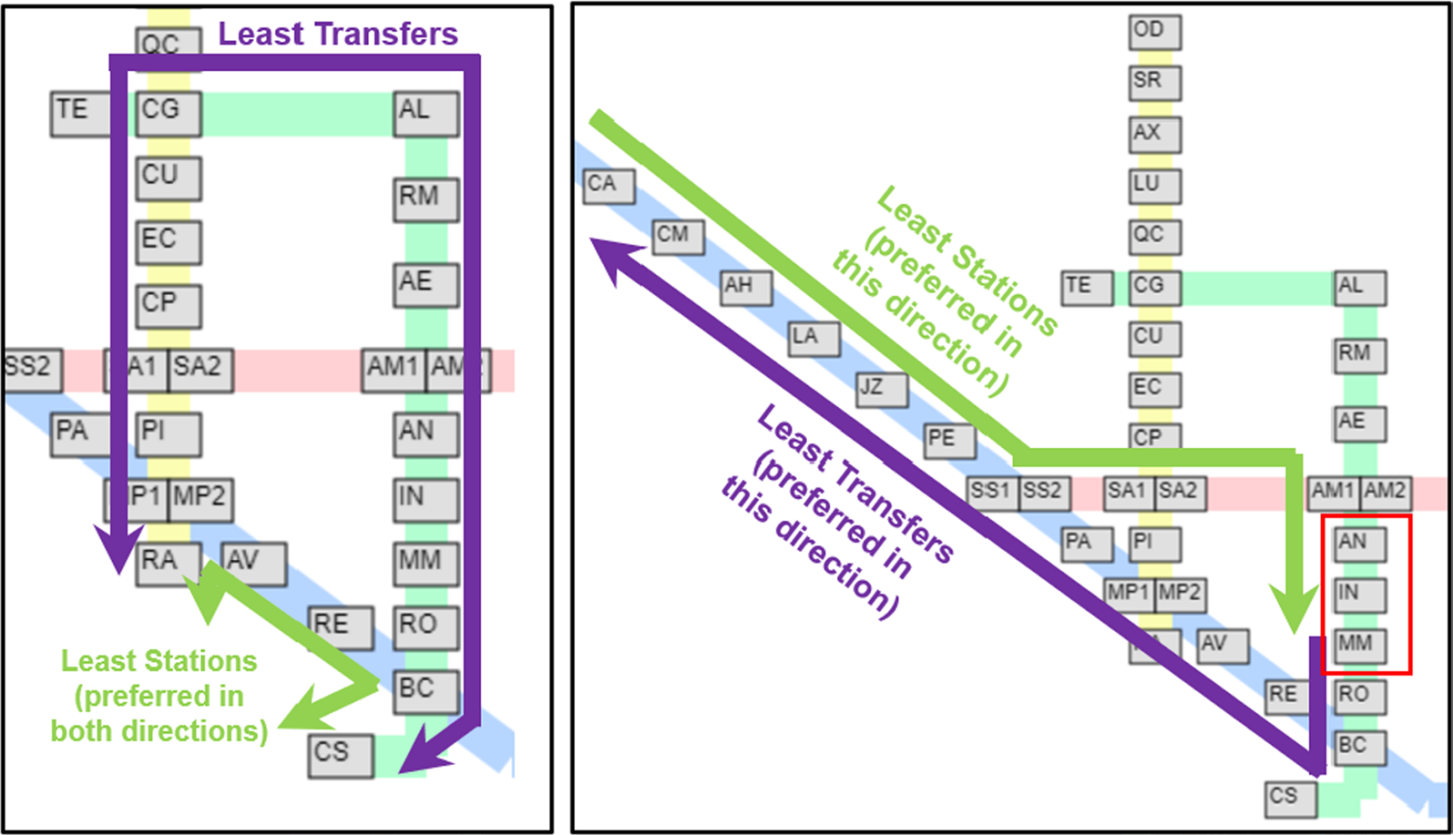
Visualization of non-trivial pair route choices. Left: Rato (RA) $\leftrightarrow$ Cais do Sodré (CS) and Right: Stations in the upper portion of the blue line $\leftrightarrow$ Anjos (AS), Intendente (IN), Martim Moniz (MM)

Figure 11 shows the preference of the passengers for station pairs between the yellow-green and blue-green lines. For the stations between the yellow-green line, it can be seen that passengers prefer the least number of transfers in most cases. However, there are noticeable exceptions on the station pairs on the lower-right portion. This tendency can be explained by the fact that in these pairs, it is possible to include an additional transfer to the red line to cut several stations from the trip. In the most extreme case, take for example the case of Rato (RA) $\leftrightarrow$ Cais do Sodré (CS), illustrated in Fig. 12. To minimize the transfers in these routes, one has to do one transfer at Campo Grande (CG). However, it is possible to do two transfers at Marquês de Pombal (MP1/MP2) and Baixa-Chiado (BC) to cut the trip much shorter. As the tradeoff is quite large, it is reasonable that a significant amount of passengers preferred the route with more transfers.

For the stations between the blue-green line, once again most passengers preferred routes with least transfers. However, a significant amount of passengers going to Anjos (AN), Intendente (IN), and Martim Moniz (MM) from the northern blue line stations tend to pick the route with the least stations. Interestingly, however, this was not case for the reverse direction, as also illustrated in Fig. 12. A possible explanation for this is the flow of the passengers throughout the day. The area around AN, IN, and MM stations is mostly commercial, and based on the volume of entrance-exit pairs, it is known that the passenger flow goes from the northern blue line stations to the commercial areas in the morning and in the reverse direction at night. It is possible that variations in route behavior are influenced by factors such as the desire to be on-time in the morning, while convenience is more prioritized at the end of the day.

**Fig. 13:**
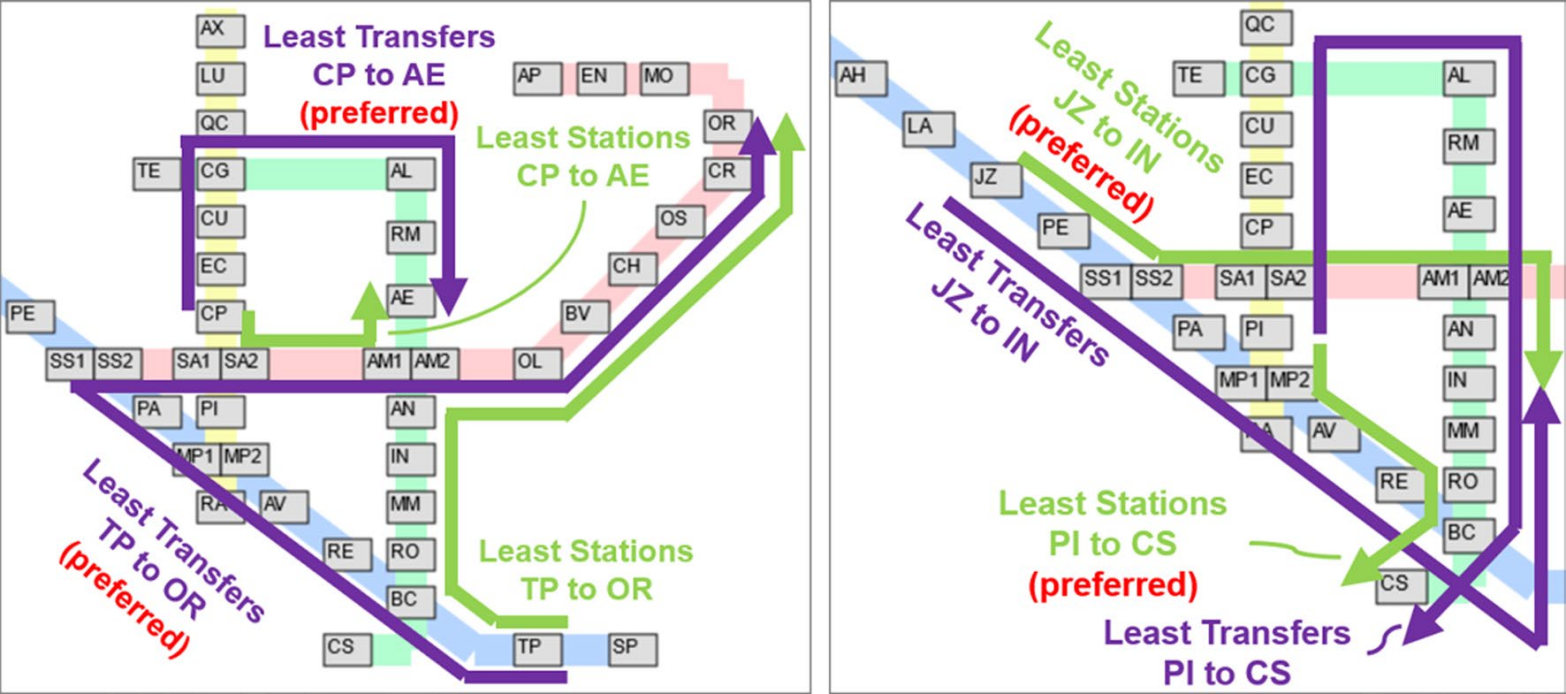
Visualizations of examples (b) and (c) where the route with least transfers is preferred (left) and examples (e) and (g) where the route with least stations is preferred (right)

Table 2 shows more selected cases among non-trivial pairs. In examples (a–d), the route with the least transfers was the preferred route, while in examples (e–g) the preference is towards routes with the least number of stations. As shown in Fig. 13, for passengers travelling from Campo Pequeno (CP) to Areeiro (AE), more passengers preferred to take the route with only one transfer, even it had a longer distance. However, this is not always the case, as for passengers travelling from Jardim Zoologico (JZ) to Intendente (IN), slightly more passengers took the route with more transfers but with lower distance, as discussed previously. This shows the variability of passenger preferences as influenced by a variety of hidden factors that cannot be straightforwardly captured by deterministic models.

**Table 2:** Some examples among non-trivial pair

	Entry station	Exit station	Least transfers route	Ratio	Least distance route	Ratio
(a)	AN	AS	AN$\rightarrow$ BC$\rightarrow$ AS	**0.98**	AN$\rightarrow$ AM$\rightarrow$ SS$\rightarrow$ AS	0.02
			(1 transfer, 18 stations)		(2 transfers, 12 stations)	
(b)	TP	OR	TP$\rightarrow$ SS$\rightarrow$ OR	**0.96**	TP$\rightarrow$ BC$\rightarrow$ AM$\rightarrow$ OR	0.02
			(1 transfer, 14 stations)		(2 transfers, 12 stations)	
c)	CP	AE	CP$\rightarrow$ CG$\rightarrow$ AE	**0.56**	CP$\rightarrow$ SA$\rightarrow$ AM$\rightarrow$ AE	0.44
			(1 transfer, 6 stations)		(2 transfers, 3 stations)	
(d)	AM	AV	AM$\rightarrow$ SS$\rightarrow$ AV	**0.28**	AM$\rightarrow$ SA$\rightarrow$ MP$\rightarrow$ AV	0.01
			(1 transfer, 6 stations)		(2 transfers, 4 stations)	
			AM$\rightarrow$ BC$\rightarrow$ AV	**0.70**		
			(1 transfer, 7 stations)			
(e)	PI	CS	PI$\rightarrow$ CG$\rightarrow$ CS	0.11	PI$\rightarrow$ MP$\rightarrow$ BC$\rightarrow$ CS	**0.86**
			(1 transfer, 15 stations)		(2 transfers, 5 stations)	
(f)	CS	PI	CS$\rightarrow$ CG$\rightarrow$ PI	0.06	CS$\rightarrow$ BC$\rightarrow$ MP$\rightarrow$ PI	**0.57**
			(1 transfer, 15 stations)		(2 transfers, 5 stations)	
(g)	JZ	IN	JZ$\rightarrow$ BC$\rightarrow$ IN	0.47	JZ$\rightarrow$ SS$\rightarrow$ AM$\rightarrow$ IN	**0.51**
			(1 transfer, 10 stations)		(2 transfers, 6 stations)	

Bold values indicate larger percentage

**Fig. 14:**
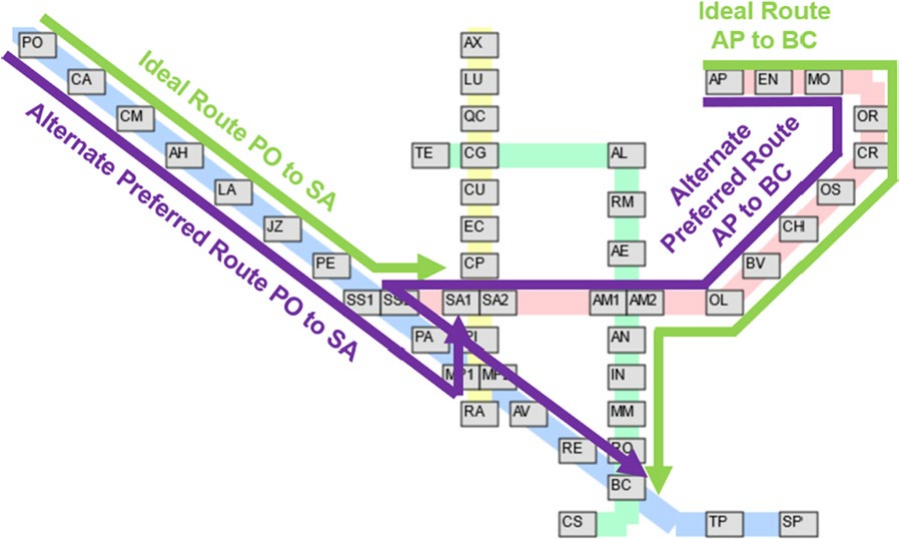
Visualizations of selected examples (h) and (k)

Finally, examining the trivial pairs where the route with the least transfers is also the route with the least stations, that route is the preferred route choice in almost all cases as to be expected. However, there are cases where evidence suggests this is not the case. We present two cases of this in Fig. 14. In the left example, although the ideal route to Saldanha (SA) from various stations in the northern half of the blue line is through a transfer in São Sebastião (SS), relatively higher number of individuals preferred to transfer in Marquês de Pombal (MP) instead. This observation can be partially explained by the large walking distance that is associated with the Alameda’s transfer. Similarly, in examples (k) and (l), a comparable number of passengers chose to transfer in São Sebastião (SS) instead of Alameda (AM) on the way to Baixa-Chiado (BC). These examples demonstrate instances where the predicted route choice contradicts the obvious choice and may be of interest to administrators and stakeholders of the metro.

Overall, these results show that the proposed approach is able to make predictions of passenger route choices in urban rail transit systems that is consistent with background expectations, while at the same time revealing interesting phenomena on passenger behavior in certain instances.

We highlight three main strengths of the proposed approach in comparison with other works on passenger route choice inference: (a) non-requirement of precise train timetables, (b) robustness to short-term changes, and (c) consideration of missed or skipped trains.

First, the non-requirement of precise train timetables means that our approach can infer passenger route choices even without knowing the exact times the trains arrived at each station. This is important because not all urban rail systems around the world follow fixed schedules that are observed to the dot. In the context of our case study on the Lisbon’s metro, train schedules are not precise/fixed. Nonetheless, estimated waiting times between each train are defined for each line at different times of the day. Still, these intervals are only estimates and are generally not followed on the dot. One of the main contributions of our work is to infer the arrival times of trains in each station even in the absence of timetables, which is a required input in previous studies [8, 10, 18, 21, 23].

To assess the importance of estimating train locations in the absence of precise timetables, we compared the proposed solution against one based on the theoretical waiting time intervals from the Lisbon metro website.^2^ For instance, according to the metro website, the waiting time for the blue line on weekdays from 10:01 a.m. to 4:45 p.m. is 6 min and 40 s. Following this assumption, we once again used the single-route case to validate whether the actual passenger exit times coincide with the expected exit times from the train movements. We base the arrival of the first train on the first passenger volume peak at the exit gate for the station in question, then assume that succeeding trains arrive at 6 min and 40 s from this interval as indicated in the website.

**Fig. 15:**
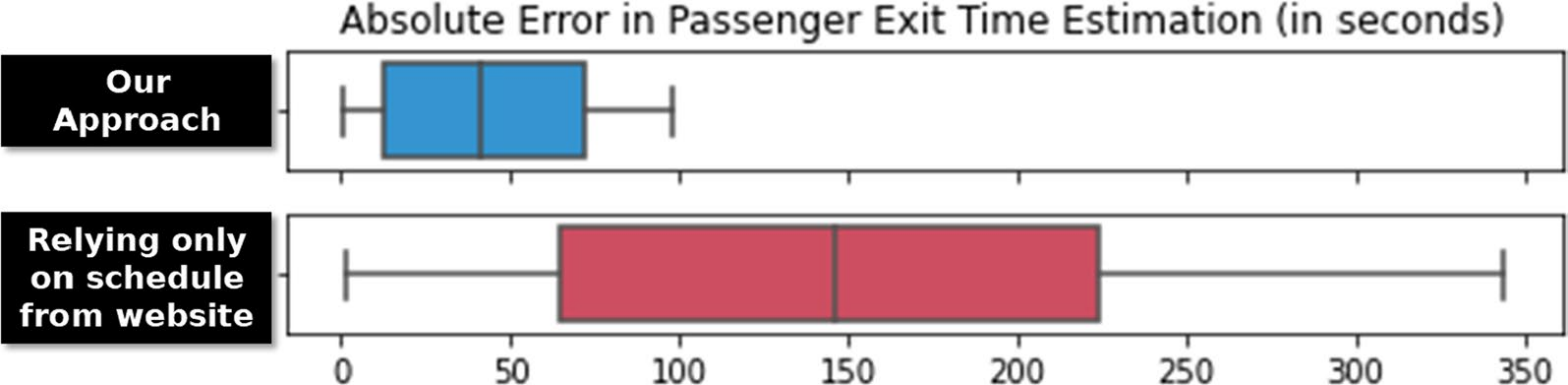
Absolute error in exit time estimation for passengers in São Sebastião $\rightarrow$ Reboleira from 10 a.m. to 2 p.m., using our approach and relying only on the operational schedule specifications, showing that our approach can estimate passenger exit times with significantly lower error ($p=0.0075$)

Figure 15 shows the distribution of the absolute error in seconds, of estimating passengers’ exit times using (a) our proposed approach, and (b) the train dispatch intervals based on the website as described above. This figure shows a one-route scenario (São Sebastião $\rightarrow$ Reboleira) from 10:00 a.m. to 2:00 p.m., where there is only one straight path from the origin and destination, eliminating possible variances in route choice. While the absence of precise timetables can still allow us to estimate passenger exit times according to operational guidelines, the estimation errors are much higher. This suggests that in the case of the Lisbon metro, the actual time intervals in-between trains do not strictly follow the prescribed values exactly. On the other hand, our approach is able to estimate the passenger exit times with a significantly lower error in a paired *t*-test ($p=0.0075$). The median error of the estimated exit times using the theoretical intervals is 146 s, while the median error of the estimated exit times using our approach is only 42 s.

**Fig. 16:**
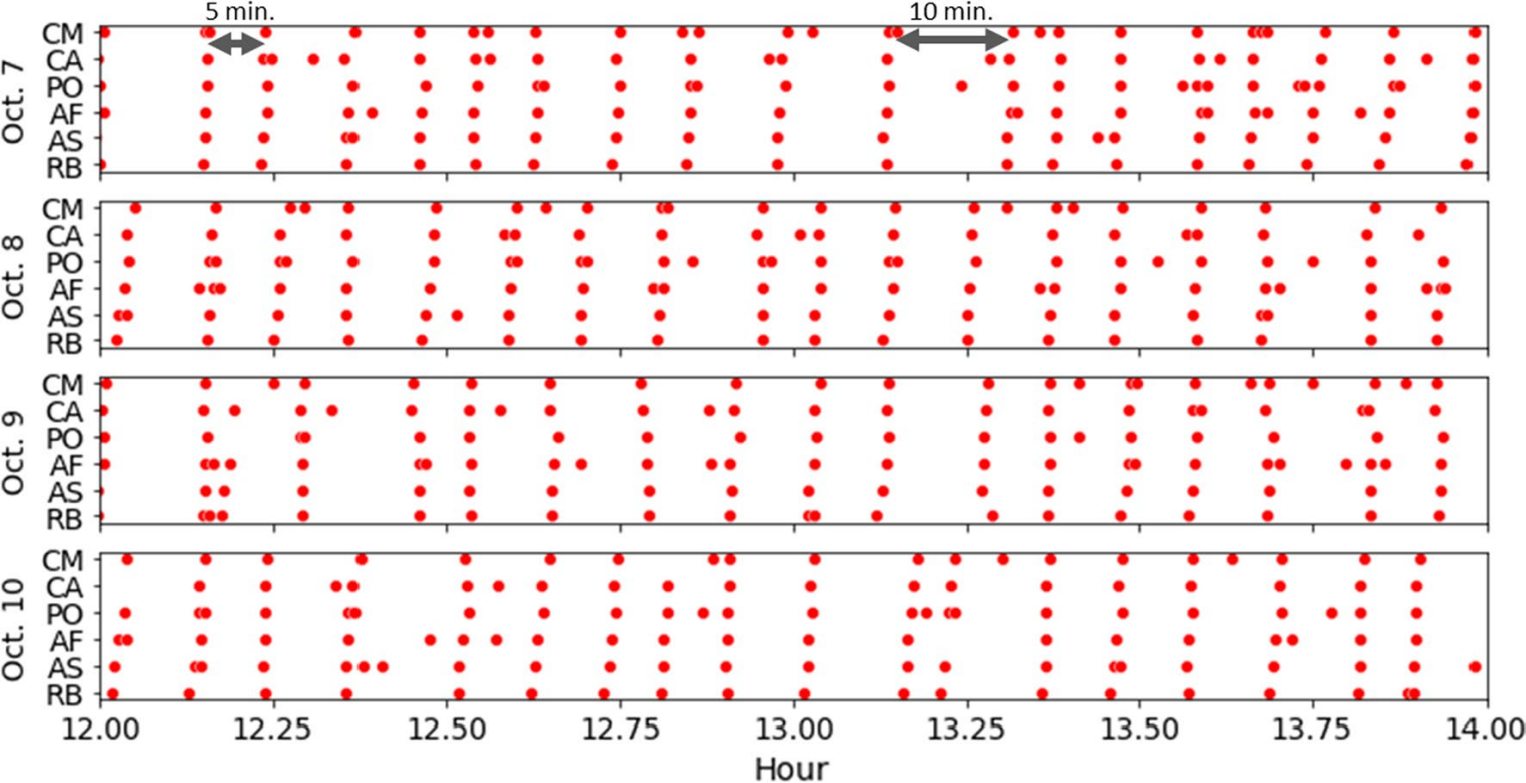
Aligned passenger volume peaks for the last six stations of the blue line for four different dates over the same time of the day, showing evidence of inconsistencies in actual train dispatch intervals

We further highlight the inconsistency of the real-world operating schedules in Fig. 16, which shows the aligned passenger volume peaks on the last six stations of the blue line for four consecutive days. Given the timeline of 12:00– 2:00 p.m., we expect that the intervals between trains are 6 min and 40 s as stated in the operational schedules. However, the aligned passenger volume peaks show clear evidence that this is not the case. In October 7 alone, the peaks indicate that the actual intervals are not consistent and could take up to 10 min. Furthermore, the intervals are not consistent across different days, despite all being weekdays. This highlights the second strength of our proposed approach, which is the robustness to short-term changes in terms of operational schedules. While previous studies rely on static timetables for train movements, our approach estimates them according to the available real-world data.

Finally, our approach also considers the possibility of passengers missing or skipping the next immediately available train. For example, in the same single-route of São Sebastião $\rightarrow$ Reboleira, 85% of the trips made on October 7, 2019 that were within 180 s of the predicted exit times fell under the condition of having no skips ($lag=0$). On the other hand, the remaining trips fell under the condition of having skipped one train ($lag=1$). These likely comprise of people who had to skip a train due to the time of walking from entrance to platform or due to other reasons such as overcrowding or personal decisions. In some previous works [8, 10], this possibility of skipping trains is not considered, which could limit the potential to infer route choices under these scenarios.

## Conclusion

In this paper, we proposed a novel approach for predicting passenger route choices in a transport network using automated fare collection data. In contrast with state-of-the-art principles on route estimation, the proposed method does not require precise train timetables and is parameterizable to contemplate the missing of trains due to arbitrarily-high movement from gate to the platform and unavailable capacity at vehicles at peak hours. The proposed method is further robust to unforeseen events, such as malfunctions and operational delays, and dynamically adaptable to topological changes in the network as long as those are standardly captured in the reference General Transit Feed Specification source.

We applied the approach to a real trip record data collected from the Lisbon metropolitan system and found that different users place different choices along the same entry-exit station pairs. Although the majority of preferred routes along the network corresponds to those with the least transfers, a considerable amount of choices do not follow this assumption. In our manuscript, we comprehensively present cases wherein the route choice by the majority of passengers is guided by the shortest distance and not by the number of transfers.

We believe that these are valuable to metro administrators and urban policymakers, as it gives a better understanding of line demand, and in-vehicle occupation levels. Inferring passenger route choices is essential to understanding the passenger flow and volume within an urban rail network, which can be used to guide a variety of aspects, such as bottleneck detection, simulation studies on railway networks (e.g., how does the deactivation of a segment affect the operations of a network), satisfaction of safety norms especially in times of a pandemic, amenity and service placements, among others. The fine-grained location of passengers along a urban rail transit systems can also support vehicle scheduling and their re-capacitation along different periods, as well as guide complementary initiatives for the positive conditioning of route choices.

Future directions on this study include supplementary ground-truth based validations against survey data, and the possibility of assessing the proposed approach on complementary case studies.

## Data Availability

The source code supporting the conclusions of this article is available in a Github repository https://github.com/thomastiamleept/metro. The source code is platform independent and written in Python 3, and is licensed under the GNU General Public License.

## Footnotes

1 https://www.metrolisboa.pt/en/travel/timetables-and-frequency%20/.

2 https://www.metrolisboa.pt/en/travel/timetables-and-frequency/.
